# Supplementary material for: Atomic protein structure refinement using all-atom graph representations and SE(3)-equivariant graph transformer
Source: Bioinformatics. 2023 May 5;39(5):btad298. doi: 10.1093/bioinformatics/btad298 (PMC10191610; doi:10.1093/bioinformatics/btad298)
Supplement: btad298_Supplementary_Data [file btad298_supplementary_data.docx]

**Table S1.** List of all atom-level and backbone atom-level features.

| Methods | Feature Type | Feature Name | Details |
| --- | --- | --- | --- |
| All atom-level features  for ATOMRefine | Node feature | Atom Embedding | 37 atom types (N, CA, C, O, CB, OG, CG, CD1, CD2, CE1, CE2, CZ, OD1, ND2, CG1, CG2, CD, CE, NZ, OD2, OE1, NE2, OE2, OH, NE, NH1, NH2, OG1, SD, ND1, SG, NE1, CE3, CZ2, CZ3, CH2, OXT) + 21 AA types (20 natural amino acids and one for unknown) |
|  | Edge feature | Distance-Based Edge Features | Inter-atom distance matrices |
|  |  | Covalent bond matrix | The distance matrix is then thresholded to entries less than this distance plus some tolerance to create and adjacency bond matrix. This adjacency bond matrix is then parsed into an edge list. |
|  |  | Relative Edge Features | Relative Position Edge Features  Relative Orientation Edge Features |
| Backbone atom-level (or residue level) features for ATOMRefine_backbone | Node feature | Amino Acid Embedding | One-hot encoding of 21 AA types (20 natural amino acids and one for unknown) |
|  |  | Dihedral angle | In radian |
|  |  | Secondary structure | 3 states |
|  |  | Relative solvent accessibility | Calculated by DSSP and ranges from [0,1] |
|  | Edge feature | Distance-Based Edge Features | Distance maps (Ca - Ca, Cb - Cb and N - O) |
|  |  | Orientation features map | ω, θ, φ calculated by trRosetta |

Note: the x, y, z coordinate of atom is also a node feature that is updated by the network, which is not shown in the table above.

**Table S2.** The target-by-target performance of ATOMRefine, GNNRefine, and ModRefiner on 193 targets in AlphaFoldDB test set evaluated by GDT-HA.

| Target ID | initial model | ATOMRefine | | | ATOMRefine_backbone | | | GNNRefine | | | ModRefiner | | |
| --- | --- | --- | --- | --- | --- | --- | --- | --- | --- | --- | --- | --- | --- |
|  |  | Mean | Minimum | Maximum | Mean | Minimum | Maximum | Mean | Minimum | Maximum | Mean | Minimum | Maximum |
| 1C8UA | 0.8842 | 0.8807 | 0.8781 | 0.8833 | 0.8847 | 0.8825 | 0.8868 | 0.8319 | 0.7956 | 0.8658 | 0.8702 | 0.8667 | 0.8763 |
| 1D4VB | 0.8666 | 0.8678 | 0.8666 | 0.8681 | 0.8568 | 0.8512 | 0.8650 | 0.8374 | 0.8067 | 0.8650 | 0.8647 | 0.8604 | 0.8696 |
| 1FWUA | 0.9198 | 0.9175 | 0.9160 | 0.9198 | 0.9217 | 0.9179 | 0.9254 | 0.8989 | 0.8750 | 0.9179 | 0.9011 | 0.8843 | 0.9086 |
| 1GO4E | 0.3764 | 0.3822 | 0.3764 | 0.3879 | 0.3787 | 0.3736 | 0.3822 | 0.3839 | 0.3649 | 0.3937 | 0.3782 | 0.3707 | 0.3822 |
| 1GYUA | 0.9433 | 0.9416 | 0.9412 | 0.9433 | 0.9420 | 0.9370 | 0.9454 | 0.8819 | 0.8613 | 0.9076 | 0.9320 | 0.9223 | 0.9391 |
| 1HN6A | 0.1364 | 0.1368 | 0.1364 | 0.1386 | 0.1405 | 0.1364 | 0.1432 | 0.1382 | 0.1364 | 0.1409 | 0.1364 | 0.1364 | 0.1364 |
| 1J26A | 0.6250 | 0.6180 | 0.6150 | 0.6200 | 0.6240 | 0.6200 | 0.6275 | 0.6210 | 0.6025 | 0.6300 | 0.6250 | 0.6225 | 0.6275 |
| 1J8CA | 0.4296 | 0.4301 | 0.4223 | 0.4393 | 0.4311 | 0.4175 | 0.4417 | 0.4049 | 0.3981 | 0.4150 | 0.4277 | 0.4223 | 0.4393 |
| 1J8IA | 0.4462 | 0.4398 | 0.4328 | 0.4435 | 0.4425 | 0.4409 | 0.4435 | 0.4441 | 0.4382 | 0.4489 | 0.4462 | 0.4382 | 0.4516 |
| 1JQLB | 0.7946 | 0.7868 | 0.7786 | 0.7946 | 0.7943 | 0.7911 | 0.8000 | 0.7704 | 0.7536 | 0.7768 | 0.7893 | 0.7857 | 0.7964 |
| 1JU5A | 0.5550 | 0.5523 | 0.5505 | 0.5550 | 0.5596 | 0.5550 | 0.5619 | 0.5568 | 0.5550 | 0.5596 | 0.5541 | 0.5436 | 0.5619 |
| 1K8IB | 0.8553 | 0.8523 | 0.8513 | 0.8539 | 0.8479 | 0.8382 | 0.8513 | 0.7240 | 0.5987 | 0.7842 | 0.8421 | 0.8342 | 0.8461 |
| 1KQGB | 0.8218 | 0.8187 | 0.8175 | 0.8209 | 0.8109 | 0.8054 | 0.8183 | 0.7362 | 0.6990 | 0.7708 | 0.8083 | 0.8019 | 0.8149 |
| 1LFBA | 0.8669 | 0.8662 | 0.8636 | 0.8701 | 0.8740 | 0.8701 | 0.8766 | 0.8448 | 0.8312 | 0.8571 | 0.8669 | 0.8636 | 0.8701 |
| 1LR5B | 0.9780 | 0.9786 | 0.9780 | 0.9796 | 0.9748 | 0.9733 | 0.9764 | 0.9412 | 0.9198 | 0.9607 | 0.9745 | 0.9717 | 0.9764 |
| 1N54B | 0.7975 | 0.7996 | 0.7975 | 0.8037 | 0.7967 | 0.7955 | 0.7975 | 0.7789 | 0.7707 | 0.7913 | 0.7938 | 0.7913 | 0.7975 |
| 1NFIE | 0.8950 | 0.8872 | 0.8785 | 0.8950 | 0.8941 | 0.8892 | 0.8986 | 0.8337 | 0.7830 | 0.8573 | 0.8604 | 0.8526 | 0.8703 |
| 1O8RA | 0.3856 | 0.3856 | 0.3777 | 0.3883 | 0.3824 | 0.3723 | 0.3883 | 0.3830 | 0.3803 | 0.3856 | 0.3846 | 0.3777 | 0.3883 |
| 1OYJC | 0.8491 | 0.8480 | 0.8447 | 0.8513 | 0.8445 | 0.8414 | 0.8480 | 0.8359 | 0.8128 | 0.8447 | 0.8238 | 0.8106 | 0.8326 |
| 1P1TA | 0.7019 | 0.6985 | 0.6971 | 0.7019 | 0.7033 | 0.6995 | 0.7091 | 0.6553 | 0.6442 | 0.6707 | 0.6985 | 0.6947 | 0.7067 |
| 1PF5A | 0.9269 | 0.9250 | 0.9212 | 0.9288 | 0.9288 | 0.9269 | 0.9327 | 0.8554 | 0.8077 | 0.8981 | 0.9123 | 0.9077 | 0.9192 |
| 1Q6QB | 0.9343 | 0.9317 | 0.9284 | 0.9343 | 0.9369 | 0.9343 | 0.9390 | 0.9223 | 0.9155 | 0.9272 | 0.9195 | 0.9002 | 0.9331 |
| 1R8UA | 0.4500 | 0.4430 | 0.4350 | 0.4550 | 0.4440 | 0.4200 | 0.4550 | 0.3790 | 0.3700 | 0.3900 | 0.4540 | 0.4450 | 0.4600 |
| 1SR3A | 0.6689 | 0.6667 | 0.6645 | 0.6711 | 0.6698 | 0.6689 | 0.6711 | 0.6544 | 0.6382 | 0.6667 | 0.6605 | 0.6491 | 0.6667 |
| 1SRAA | 0.9470 | 0.9493 | 0.9470 | 0.9536 | 0.9483 | 0.9454 | 0.9503 | 0.9315 | 0.9123 | 0.9454 | 0.9215 | 0.9123 | 0.9305 |
| 1TNRA | 0.9722 | 0.9750 | 0.9740 | 0.9757 | 0.9702 | 0.9688 | 0.9705 | 0.9479 | 0.9306 | 0.9653 | 0.9573 | 0.9531 | 0.9653 |
| 1VI7A | 0.7083 | 0.7074 | 0.7059 | 0.7096 | 0.7064 | 0.7034 | 0.7083 | 0.6681 | 0.6385 | 0.6789 | 0.6946 | 0.6875 | 0.7047 |
| 1WJPA | 0.3059 | 0.3032 | 0.3005 | 0.3059 | 0.3037 | 0.3032 | 0.3059 | 0.2963 | 0.2872 | 0.3085 | 0.3117 | 0.3059 | 0.3165 |
| 1WOUA | 0.9328 | 0.9345 | 0.9307 | 0.9370 | 0.9328 | 0.9307 | 0.9349 | 0.9227 | 0.9118 | 0.9328 | 0.9269 | 0.9223 | 0.9307 |
| 1WSPB | 0.8512 | 0.8434 | 0.8393 | 0.8452 | 0.8429 | 0.8393 | 0.8452 | 0.7952 | 0.7857 | 0.8155 | 0.8393 | 0.8304 | 0.8452 |
| 1XJHA | 0.5984 | 0.6033 | 0.5984 | 0.6066 | 0.5976 | 0.5943 | 0.6025 | 0.6344 | 0.6270 | 0.6393 | 0.6041 | 0.5902 | 0.6107 |
| 1YXMA | 0.8333 | 0.8323 | 0.8308 | 0.8350 | 0.8342 | 0.8316 | 0.8359 | 0.7946 | 0.7744 | 0.8064 | 0.8059 | 0.7988 | 0.8224 |
| 1Z6UA | 0.9500 | 0.9513 | 0.9500 | 0.9543 | 0.9470 | 0.9413 | 0.9500 | 0.9448 | 0.9196 | 0.9609 | 0.9439 | 0.9391 | 0.9522 |
| 1ZZAA | 0.2841 | 0.2864 | 0.2841 | 0.2898 | 0.2687 | 0.2585 | 0.2813 | 0.2779 | 0.2727 | 0.2841 | 0.2847 | 0.2813 | 0.2869 |
| 2AKAB | 0.7391 | 0.7323 | 0.7308 | 0.7333 | 0.7366 | 0.7349 | 0.7391 | 0.7227 | 0.7040 | 0.7400 | 0.7284 | 0.7216 | 0.7366 |
| 2AL3A | 0.4704 | 0.4684 | 0.4671 | 0.4704 | 0.4717 | 0.4704 | 0.4737 | 0.4776 | 0.4671 | 0.4868 | 0.4777 | 0.4704 | 0.4836 |
| 2C3NA | 0.9561 | 0.9569 | 0.9550 | 0.9582 | 0.9586 | 0.9561 | 0.9613 | 0.9477 | 0.9299 | 0.9592 | 0.9318 | 0.9163 | 0.9435 |
| 2CZOA | 0.5346 | 0.5327 | 0.5288 | 0.5385 | 0.5346 | 0.5327 | 0.5365 | 0.5285 | 0.5231 | 0.5346 | 0.5319 | 0.5269 | 0.5365 |
| 2EBMA | 0.7831 | 0.7835 | 0.7810 | 0.7872 | 0.7839 | 0.7810 | 0.7872 | 0.7351 | 0.7190 | 0.7438 | 0.7748 | 0.7645 | 0.7831 |
| 2EE7A | 0.6354 | 0.6392 | 0.6354 | 0.6417 | 0.6338 | 0.6271 | 0.6396 | 0.6329 | 0.6292 | 0.6354 | 0.6338 | 0.6271 | 0.6417 |
| 2EKFA | 0.6574 | 0.6518 | 0.6481 | 0.6574 | 0.6518 | 0.6481 | 0.6574 | 0.6315 | 0.6250 | 0.6343 | 0.6565 | 0.6528 | 0.6574 |
| 2FR2A | 0.9441 | 0.9416 | 0.9394 | 0.9457 | 0.9413 | 0.9379 | 0.9441 | 0.9140 | 0.9053 | 0.9208 | 0.9391 | 0.9270 | 0.9565 |
| 2G9BA | 0.3697 | 0.3678 | 0.3649 | 0.3716 | 0.3695 | 0.3678 | 0.3716 | 0.3705 | 0.3630 | 0.3764 | 0.3724 | 0.3688 | 0.3755 |
| 2GCUB | 0.9839 | 0.9820 | 0.9818 | 0.9828 | 0.9820 | 0.9807 | 0.9839 | 0.9455 | 0.9313 | 0.9624 | 0.9691 | 0.9678 | 0.9710 |
| 2HNTE | 0.8209 | 0.8179 | 0.8022 | 0.8284 | 0.8097 | 0.7761 | 0.8246 | 0.5366 | 0.5112 | 0.5597 | 0.8239 | 0.8209 | 0.8284 |
| 2JO6A | 0.7315 | 0.7236 | 0.7222 | 0.7292 | 0.7241 | 0.7176 | 0.7292 | 0.7009 | 0.6759 | 0.7153 | 0.7338 | 0.7292 | 0.7431 |
| 2JQLA | 0.4823 | 0.4745 | 0.4716 | 0.4752 | 0.4731 | 0.4628 | 0.4770 | 0.4770 | 0.4699 | 0.4840 | 0.4801 | 0.4734 | 0.4876 |
| 2JT4A | 0.7183 | 0.7190 | 0.7148 | 0.7218 | 0.7197 | 0.7148 | 0.7254 | 0.7035 | 0.6937 | 0.7183 | 0.7176 | 0.7113 | 0.7254 |
| 2JTSA | 0.8588 | 0.8488 | 0.8412 | 0.8529 | 0.8506 | 0.8471 | 0.8529 | 0.8429 | 0.8265 | 0.8529 | 0.8565 | 0.8529 | 0.8618 |
| 2K4FA | 0.1652 | 0.1643 | 0.1563 | 0.1696 | 0.1268 | 0.0938 | 0.1563 | 0.1286 | 0.1205 | 0.1384 | 0.1670 | 0.1652 | 0.1696 |
| 2KL7A | 0.1786 | 0.1772 | 0.1750 | 0.1786 | 0.1729 | 0.1679 | 0.1786 | 0.1793 | 0.1786 | 0.1821 | 0.1772 | 0.1750 | 0.1786 |
| 2KUEA | 0.3759 | 0.3756 | 0.3741 | 0.3778 | 0.3800 | 0.3796 | 0.3815 | 0.3804 | 0.3741 | 0.3852 | 0.3685 | 0.3648 | 0.3741 |
| 2KVCA | 0.5725 | 0.5670 | 0.5650 | 0.5700 | 0.5715 | 0.5700 | 0.5725 | 0.5715 | 0.5650 | 0.5800 | 0.5770 | 0.5675 | 0.5850 |
| 2KZ3A | 0.3072 | 0.3078 | 0.3042 | 0.3133 | 0.3084 | 0.3072 | 0.3102 | 0.3133 | 0.3072 | 0.3193 | 0.3090 | 0.3042 | 0.3133 |
| 2L9QA | 0.2271 | 0.2266 | 0.2248 | 0.2271 | 0.2248 | 0.2225 | 0.2271 | 0.2298 | 0.2248 | 0.2339 | 0.2285 | 0.2271 | 0.2317 |
| 2LCLA | 0.1694 | 0.1661 | 0.1653 | 0.1694 | 0.1605 | 0.1532 | 0.1694 | 0.1669 | 0.1653 | 0.1694 | 0.1678 | 0.1653 | 0.1694 |
| 2LLZA | 0.6633 | 0.6571 | 0.6505 | 0.6607 | 0.6607 | 0.6556 | 0.6658 | 0.6516 | 0.6327 | 0.6684 | 0.6745 | 0.6684 | 0.6811 |
| 2LRVA | 0.5327 | 0.5280 | 0.5238 | 0.5298 | 0.5322 | 0.5268 | 0.5387 | 0.5262 | 0.5179 | 0.5357 | 0.5286 | 0.5238 | 0.5327 |
| 2LXEA | 0.4017 | 0.4039 | 0.4017 | 0.4045 | 0.4034 | 0.3989 | 0.4073 | 0.4067 | 0.4017 | 0.4129 | 0.4023 | 0.3961 | 0.4073 |
| 2M0RA | 0.4447 | 0.4490 | 0.4447 | 0.4519 | 0.4096 | 0.3894 | 0.4279 | 0.4423 | 0.4375 | 0.4471 | 0.4442 | 0.4375 | 0.4519 |
| 2MI2A | 0.4629 | 0.4609 | 0.4554 | 0.4653 | 0.4619 | 0.4579 | 0.4678 | 0.2683 | 0.2649 | 0.2748 | 0.4550 | 0.4505 | 0.4604 |
| 2MXYA | 0.6857 | 0.6838 | 0.6762 | 0.6881 | 0.6738 | 0.6619 | 0.6857 | 0.6829 | 0.6786 | 0.6905 | 0.6938 | 0.6905 | 0.7000 |
| 2MZBA | 0.5497 | 0.5485 | 0.5472 | 0.5523 | 0.5518 | 0.5485 | 0.5548 | 0.5508 | 0.5434 | 0.5561 | 0.5444 | 0.5421 | 0.5472 |
| 2NLWA | 0.4500 | 0.4548 | 0.4524 | 0.4571 | 0.4519 | 0.4476 | 0.4548 | 0.4272 | 0.4214 | 0.4310 | 0.4510 | 0.4452 | 0.4548 |
| 2PJYA | 0.6362 | 0.6357 | 0.6317 | 0.6384 | 0.6402 | 0.6362 | 0.6429 | 0.6223 | 0.6116 | 0.6339 | 0.6281 | 0.6250 | 0.6295 |
| 2PKPA | 0.8413 | 0.8392 | 0.8368 | 0.8428 | 0.8431 | 0.8413 | 0.8458 | 0.8377 | 0.8263 | 0.8443 | 0.8249 | 0.8189 | 0.8293 |
| 2PNGA | 0.4101 | 0.4135 | 0.4101 | 0.4157 | 0.4219 | 0.4185 | 0.4242 | 0.4039 | 0.3961 | 0.4129 | 0.4123 | 0.4073 | 0.4157 |
| 2RMSB | 0.2787 | 0.2787 | 0.2746 | 0.2828 | 0.2385 | 0.1721 | 0.2828 | 0.2828 | 0.2787 | 0.2869 | 0.2853 | 0.2828 | 0.2869 |
| 2RT9A | 0.7356 | 0.7366 | 0.7308 | 0.7452 | 0.7346 | 0.7308 | 0.7404 | 0.6923 | 0.6731 | 0.7115 | 0.7318 | 0.7212 | 0.7356 |
| 2VK2A | 0.9966 | 0.9958 | 0.9949 | 0.9966 | 0.9963 | 0.9949 | 0.9975 | 0.9465 | 0.8995 | 0.9806 | 0.9848 | 0.9806 | 0.9882 |
| 2VZZA | 0.9405 | 0.9414 | 0.9393 | 0.9440 | 0.9407 | 0.9393 | 0.9417 | 0.8872 | 0.8417 | 0.9202 | 0.9079 | 0.8857 | 0.9214 |
| 2XVSA | 0.8567 | 0.8564 | 0.8521 | 0.8598 | 0.8558 | 0.8521 | 0.8598 | 0.8476 | 0.8262 | 0.8643 | 0.8476 | 0.8384 | 0.8552 |
| 2YWJA | 0.8135 | 0.8106 | 0.8014 | 0.8149 | 0.8138 | 0.8122 | 0.8162 | 0.8060 | 0.7784 | 0.8230 | 0.8095 | 0.8054 | 0.8122 |
| 2Z84A | 0.9557 | 0.9543 | 0.9522 | 0.9569 | 0.9531 | 0.9498 | 0.9557 | 0.9079 | 0.8589 | 0.9462 | 0.9474 | 0.9474 | 0.9474 |
| 2Z9DA | 0.9588 | 0.9545 | 0.9488 | 0.9588 | 0.9585 | 0.9563 | 0.9600 | 0.9370 | 0.9287 | 0.9463 | 0.9285 | 0.9175 | 0.9425 |
| 3BCIA | 0.9879 | 0.9858 | 0.9833 | 0.9894 | 0.9882 | 0.9879 | 0.9894 | 0.9179 | 0.8803 | 0.9379 | 0.9791 | 0.9697 | 0.9833 |
| 3C90X | 0.9511 | 0.9540 | 0.9483 | 0.9569 | 0.9517 | 0.9483 | 0.9569 | 0.9046 | 0.8937 | 0.9167 | 0.9477 | 0.9425 | 0.9511 |
| 3COQA | 0.4354 | 0.4326 | 0.4298 | 0.4354 | 0.4315 | 0.4298 | 0.4354 | 0.3798 | 0.3764 | 0.3904 | 0.4304 | 0.4242 | 0.4354 |
| 3CXHE | 0.6986 | 0.6967 | 0.6946 | 0.7000 | 0.6995 | 0.6959 | 0.7014 | 0.6584 | 0.6527 | 0.6649 | 0.6894 | 0.6838 | 0.6973 |
| 3E3UA | 0.9171 | 0.9148 | 0.9120 | 0.9171 | 0.9178 | 0.9120 | 0.9209 | 0.8857 | 0.8610 | 0.9031 | 0.9074 | 0.9031 | 0.9107 |
| 3FGTA | 0.9722 | 0.9667 | 0.9625 | 0.9694 | 0.9725 | 0.9708 | 0.9736 | 0.8819 | 0.8111 | 0.9292 | 0.9631 | 0.9583 | 0.9681 |
| 3G7EA | 0.7919 | 0.7934 | 0.7894 | 0.7993 | 0.7951 | 0.7931 | 0.7993 | 0.7641 | 0.7537 | 0.7796 | 0.7810 | 0.7734 | 0.7857 |
| 3HTKC | 0.6122 | 0.6114 | 0.6093 | 0.6132 | 0.6100 | 0.6073 | 0.6122 | 0.5638 | 0.5571 | 0.5748 | 0.6150 | 0.6093 | 0.6230 |
| 3I9WA | 0.7537 | 0.7559 | 0.7500 | 0.7602 | 0.7550 | 0.7528 | 0.7574 | 0.7106 | 0.7046 | 0.7176 | 0.7443 | 0.7361 | 0.7556 |
| 3IX0B | 0.7979 | 0.7953 | 0.7926 | 0.7979 | 0.7931 | 0.7926 | 0.7952 | 0.7245 | 0.6915 | 0.7793 | 0.7974 | 0.7899 | 0.8059 |
| 3J16G | 0.4058 | 0.4005 | 0.3957 | 0.4033 | 0.4038 | 0.4020 | 0.4045 | 0.3832 | 0.3719 | 0.3970 | 0.4013 | 0.3970 | 0.4045 |
| 3J7YU | 0.9144 | 0.9068 | 0.8986 | 0.9167 | 0.9171 | 0.9144 | 0.9189 | 0.6811 | 0.6509 | 0.7725 | 0.9086 | 0.8896 | 0.9167 |
| 3KR3D | 0.4708 | 0.4775 | 0.4750 | 0.4833 | 0.4842 | 0.4792 | 0.4875 | 0.4875 | 0.4792 | 0.4958 | 0.4775 | 0.4708 | 0.4833 |
| 3LW6A | 0.9927 | 0.9929 | 0.9927 | 0.9938 | 0.9897 | 0.9876 | 0.9917 | 0.9402 | 0.9025 | 0.9647 | 0.9772 | 0.9751 | 0.9803 |
| 3M9GA | 0.4490 | 0.4490 | 0.4478 | 0.4502 | 0.4512 | 0.4490 | 0.4540 | 0.4885 | 0.4751 | 0.5137 | 0.4428 | 0.4366 | 0.4490 |
| 3NI0B | 0.8908 | 0.8851 | 0.8793 | 0.8937 | 0.8851 | 0.8793 | 0.8937 | 0.5966 | 0.5546 | 0.6408 | 0.8897 | 0.8822 | 0.8937 |
| 3QNTA | 0.7941 | 0.7879 | 0.7828 | 0.7920 | 0.7936 | 0.7920 | 0.7961 | 0.7877 | 0.7664 | 0.7992 | 0.7912 | 0.7869 | 0.7992 |
| 3TVJB | 0.8554 | 0.8601 | 0.8595 | 0.8616 | 0.8525 | 0.8326 | 0.8626 | 0.8234 | 0.8079 | 0.8378 | 0.8452 | 0.8399 | 0.8512 |
| 3VWUE | 0.8728 | 0.8687 | 0.8684 | 0.8699 | 0.8754 | 0.8728 | 0.8772 | 0.8582 | 0.8538 | 0.8640 | 0.8638 | 0.8553 | 0.8699 |
| 3WE3A | 0.7645 | 0.7669 | 0.7624 | 0.7707 | 0.7537 | 0.7335 | 0.7707 | 0.7595 | 0.7521 | 0.7665 | 0.7508 | 0.7459 | 0.7541 |
| 4A54A | 0.4628 | 0.4707 | 0.4654 | 0.4734 | 0.4638 | 0.4574 | 0.4707 | 0.4745 | 0.4707 | 0.4761 | 0.4638 | 0.4574 | 0.4707 |
| 4AUQB | 0.8904 | 0.8939 | 0.8860 | 0.9035 | 0.8895 | 0.8816 | 0.8947 | 0.8474 | 0.8246 | 0.8596 | 0.8912 | 0.8860 | 0.8947 |
| 4BL7A | 0.8161 | 0.8163 | 0.8137 | 0.8185 | 0.8190 | 0.8161 | 0.8233 | 0.8199 | 0.8161 | 0.8257 | 0.8053 | 0.8017 | 0.8101 |
| 4D69A | 0.9658 | 0.9635 | 0.9615 | 0.9658 | 0.9639 | 0.9626 | 0.9658 | 0.9434 | 0.9167 | 0.9573 | 0.9556 | 0.9519 | 0.9605 |
| 4FTXA | 0.6835 | 0.6823 | 0.6804 | 0.6851 | 0.6807 | 0.6772 | 0.6820 | 0.6804 | 0.6693 | 0.6851 | 0.6788 | 0.6725 | 0.6835 |
| 4JIXB | 0.8293 | 0.8240 | 0.8197 | 0.8269 | 0.8259 | 0.8245 | 0.8269 | 0.8120 | 0.8029 | 0.8197 | 0.8269 | 0.8245 | 0.8293 |
| 4JN6B | 0.9198 | 0.9166 | 0.9139 | 0.9198 | 0.9184 | 0.9172 | 0.9198 | 0.7927 | 0.7179 | 0.8539 | 0.8981 | 0.8868 | 0.9130 |
| 4JPZB | 0.5687 | 0.5697 | 0.5669 | 0.5722 | 0.5711 | 0.5634 | 0.5775 | 0.5616 | 0.5546 | 0.5687 | 0.5729 | 0.5669 | 0.5810 |
| 4JVWA | 0.8542 | 0.8602 | 0.8542 | 0.8657 | 0.8611 | 0.8565 | 0.8634 | 0.8657 | 0.8565 | 0.8796 | 0.8504 | 0.8426 | 0.8634 |
| 4LN0C | 0.5550 | 0.5580 | 0.5550 | 0.5650 | 0.5330 | 0.4700 | 0.5700 | 0.3820 | 0.3650 | 0.4150 | 0.5590 | 0.5550 | 0.5600 |
| 4MSVA | 0.9478 | 0.9460 | 0.9442 | 0.9478 | 0.9482 | 0.9478 | 0.9496 | 0.9076 | 0.8849 | 0.9424 | 0.9424 | 0.9371 | 0.9496 |
| 4NW3A | 0.8676 | 0.8627 | 0.8529 | 0.8725 | 0.8676 | 0.8578 | 0.8725 | 0.8284 | 0.7990 | 0.8480 | 0.8656 | 0.8627 | 0.8676 |
| 4RBRA | 0.9277 | 0.9312 | 0.9277 | 0.9336 | 0.9269 | 0.9238 | 0.9297 | 0.8195 | 0.7988 | 0.8301 | 0.9102 | 0.9063 | 0.9141 |
| 4RDDA | 0.8895 | 0.8846 | 0.8839 | 0.8858 | 0.8843 | 0.8755 | 0.8886 | 0.8684 | 0.8502 | 0.8876 | 0.8799 | 0.8736 | 0.8848 |
| 4UZXA | 0.7808 | 0.7700 | 0.7654 | 0.7808 | 0.7738 | 0.7692 | 0.7808 | 0.7423 | 0.6962 | 0.7654 | 0.7769 | 0.7731 | 0.7808 |
| 4V4WW | 0.5753 | 0.5783 | 0.5753 | 0.5813 | 0.5819 | 0.5723 | 0.5873 | 0.6289 | 0.5994 | 0.6596 | 0.5825 | 0.5813 | 0.5843 |
| 4V7EU | 0.0989 | 0.0989 | 0.0989 | 0.0989 | 0.0989 | 0.0989 | 0.0989 | 0.1022 | 0.1016 | 0.1044 | 0.0994 | 0.0989 | 0.1016 |
| 4WWUD | 0.8225 | 0.8297 | 0.8261 | 0.8333 | 0.8304 | 0.8297 | 0.8333 | 0.7790 | 0.7681 | 0.7935 | 0.8145 | 0.8116 | 0.8188 |
| 4XGQA | 0.8950 | 0.8965 | 0.8950 | 0.8989 | 0.8942 | 0.8931 | 0.8950 | 0.8393 | 0.8130 | 0.8645 | 0.8870 | 0.8836 | 0.8912 |
| 4YLIE | 0.9648 | 0.9634 | 0.9614 | 0.9648 | 0.9476 | 0.9211 | 0.9597 | 0.8936 | 0.8607 | 0.9262 | 0.9590 | 0.9530 | 0.9631 |
| 4ZMIA | 0.9851 | 0.9823 | 0.9798 | 0.9840 | 0.9826 | 0.9809 | 0.9830 | 0.9338 | 0.8968 | 0.9596 | 0.9704 | 0.9681 | 0.9755 |
| 5A2FA | 0.7787 | 0.7727 | 0.7695 | 0.7764 | 0.7771 | 0.7741 | 0.7798 | 0.7794 | 0.7466 | 0.7924 | 0.7606 | 0.7557 | 0.7638 |
| 5DAHA | 0.8966 | 0.8897 | 0.8855 | 0.8925 | 0.8944 | 0.8925 | 0.8966 | 0.8408 | 0.8128 | 0.8757 | 0.8782 | 0.8743 | 0.8855 |
| 5HXDA | 0.9525 | 0.9475 | 0.9462 | 0.9494 | 0.9494 | 0.9483 | 0.9515 | 0.9127 | 0.8882 | 0.9357 | 0.9264 | 0.9241 | 0.9293 |
| 5IKFB | 0.9667 | 0.9604 | 0.9574 | 0.9630 | 0.9548 | 0.9481 | 0.9648 | 0.7337 | 0.7204 | 0.7593 | 0.9370 | 0.9278 | 0.9481 |
| 5JA4D | 0.7131 | 0.7148 | 0.7097 | 0.7198 | 0.7158 | 0.7148 | 0.7181 | 0.7319 | 0.7265 | 0.7383 | 0.6973 | 0.6946 | 0.7013 |
| 5JTNE | 0.1273 | 0.1346 | 0.1318 | 0.1364 | 0.1227 | 0.1182 | 0.1273 | 0.1218 | 0.1182 | 0.1227 | 0.1245 | 0.1227 | 0.1318 |
| 5L2WB | 0.9685 | 0.9646 | 0.9630 | 0.9685 | 0.9648 | 0.9639 | 0.9657 | 0.9070 | 0.8639 | 0.9472 | 0.9513 | 0.9435 | 0.9574 |
| 5LNSD | 0.9505 | 0.9461 | 0.9423 | 0.9505 | 0.9540 | 0.9496 | 0.9579 | 0.9150 | 0.8654 | 0.9405 | 0.9544 | 0.9451 | 0.9606 |
| 5LS0B | 0.9316 | 0.9263 | 0.9232 | 0.9316 | 0.9277 | 0.9260 | 0.9302 | 0.8983 | 0.8729 | 0.9162 | 0.9280 | 0.9218 | 0.9330 |
| 5LSJA | 0.3769 | 0.3776 | 0.3769 | 0.3794 | 0.3764 | 0.3744 | 0.3781 | 0.3480 | 0.3405 | 0.3618 | 0.3739 | 0.3693 | 0.3756 |
| 5MC9B | 0.2549 | 0.2598 | 0.2549 | 0.2647 | 0.2588 | 0.2255 | 0.2941 | 0.3804 | 0.3676 | 0.3873 | 0.2559 | 0.2353 | 0.2647 |
| 5MC9C | 0.5000 | 0.5104 | 0.5047 | 0.5189 | 0.4915 | 0.4434 | 0.5189 | 0.5736 | 0.5613 | 0.5943 | 0.5056 | 0.5000 | 0.5094 |
| 5MDUA | 0.9243 | 0.9217 | 0.9194 | 0.9227 | 0.9237 | 0.9227 | 0.9243 | 0.8704 | 0.8322 | 0.9079 | 0.9220 | 0.9194 | 0.9276 |
| 5MDXG | 0.7192 | 0.7178 | 0.7169 | 0.7203 | 0.7158 | 0.7089 | 0.7203 | 0.5906 | 0.5068 | 0.6495 | 0.6959 | 0.6884 | 0.7032 |
| 5MRFC | 0.9618 | 0.9594 | 0.9548 | 0.9608 | 0.9516 | 0.9408 | 0.9639 | 0.7544 | 0.7078 | 0.8062 | 0.9335 | 0.9116 | 0.9438 |
| 5MRFD | 0.9752 | 0.9738 | 0.9722 | 0.9752 | 0.9607 | 0.9563 | 0.9702 | 0.8030 | 0.7550 | 0.8601 | 0.9450 | 0.9306 | 0.9514 |
| 5MRFG | 0.9764 | 0.9784 | 0.9764 | 0.9797 | 0.9750 | 0.9561 | 0.9831 | 0.8284 | 0.7635 | 0.8851 | 0.9615 | 0.9459 | 0.9696 |
| 5OQQC | 0.6133 | 0.5930 | 0.5859 | 0.6016 | 0.5617 | 0.5039 | 0.5859 | 0.2555 | 0.2422 | 0.2617 | 0.6016 | 0.5938 | 0.6055 |
| 5T1OA | 0.5941 | 0.5965 | 0.5912 | 0.6000 | 0.5965 | 0.5912 | 0.6000 | 0.5853 | 0.5765 | 0.5941 | 0.5924 | 0.5882 | 0.5971 |
| 5T2AN | 0.7202 | 0.7185 | 0.7158 | 0.7217 | 0.7158 | 0.7113 | 0.7217 | 0.6170 | 0.6042 | 0.6414 | 0.7015 | 0.6949 | 0.7054 |
| 5TQSC | 0.8840 | 0.8856 | 0.8814 | 0.8892 | 0.8876 | 0.8840 | 0.8892 | 0.8706 | 0.8454 | 0.8840 | 0.8784 | 0.8686 | 0.8840 |
| 5U0Sc | 0.7395 | 0.7410 | 0.7395 | 0.7424 | 0.7395 | 0.7357 | 0.7424 | 0.6954 | 0.6768 | 0.7253 | 0.7247 | 0.7129 | 0.7329 |
| 5U0SR | 0.5459 | 0.5413 | 0.5374 | 0.5435 | 0.5476 | 0.5471 | 0.5483 | 0.5355 | 0.5229 | 0.5483 | 0.5384 | 0.5302 | 0.5459 |
| 5U77A | 0.8233 | 0.8194 | 0.8168 | 0.8211 | 0.8224 | 0.8211 | 0.8254 | 0.8009 | 0.7909 | 0.8082 | 0.8246 | 0.8168 | 0.8341 |
| 5UIVA | 0.8677 | 0.8679 | 0.8655 | 0.8700 | 0.8706 | 0.8655 | 0.8744 | 0.8296 | 0.8061 | 0.8509 | 0.8619 | 0.8520 | 0.8711 |
| 5V7QL | 0.7218 | 0.7218 | 0.7165 | 0.7271 | 0.7106 | 0.6954 | 0.7271 | 0.4412 | 0.4208 | 0.4560 | 0.6898 | 0.6725 | 0.7025 |
| 5VGZD | 0.4673 | 0.4701 | 0.4650 | 0.4766 | 0.4725 | 0.4720 | 0.4743 | 0.4636 | 0.4556 | 0.4720 | 0.4664 | 0.4603 | 0.4720 |
| 5WLEA | 0.8644 | 0.8581 | 0.8559 | 0.8602 | 0.8585 | 0.8559 | 0.8602 | 0.8576 | 0.8390 | 0.8771 | 0.8644 | 0.8602 | 0.8686 |
| 5XTBP | 0.8101 | 0.8149 | 0.8137 | 0.8161 | 0.7990 | 0.7692 | 0.8149 | 0.6212 | 0.5829 | 0.6623 | 0.7690 | 0.7632 | 0.7764 |
| 5Y32A | 0.8732 | 0.8715 | 0.8660 | 0.8756 | 0.8715 | 0.8696 | 0.8732 | 0.8840 | 0.8433 | 0.9019 | 0.8698 | 0.8660 | 0.8732 |
| 5ZALB | 0.6067 | 0.5940 | 0.5900 | 0.6000 | 0.5993 | 0.5933 | 0.6033 | 0.5887 | 0.5800 | 0.5967 | 0.5973 | 0.5867 | 0.6067 |
| 6AGBK | 0.6055 | 0.6039 | 0.6016 | 0.6055 | 0.6129 | 0.6055 | 0.6230 | 0.5856 | 0.5684 | 0.5977 | 0.6024 | 0.5977 | 0.6113 |
| 6AGOC | 0.8174 | 0.8162 | 0.8144 | 0.8174 | 0.8162 | 0.8084 | 0.8204 | 0.7488 | 0.7156 | 0.7710 | 0.7961 | 0.7859 | 0.8069 |
| 6AHRE | 0.5408 | 0.5439 | 0.5391 | 0.5476 | 0.5425 | 0.5374 | 0.5476 | 0.5194 | 0.5017 | 0.5357 | 0.5316 | 0.5289 | 0.5340 |
| 6AZ1Y | 0.9403 | 0.9313 | 0.9261 | 0.9375 | 0.9261 | 0.9119 | 0.9347 | 0.7307 | 0.6705 | 0.8182 | 0.9199 | 0.9148 | 0.9290 |
| 6AZ3I | 0.7406 | 0.7340 | 0.7300 | 0.7382 | 0.7120 | 0.6828 | 0.7323 | 0.5528 | 0.5436 | 0.5637 | 0.7262 | 0.7158 | 0.7394 |
| 6DR3A | 0.5946 | 0.5932 | 0.5912 | 0.5957 | 0.5959 | 0.5946 | 0.5968 | 0.5968 | 0.5867 | 0.6070 | 0.5978 | 0.5912 | 0.6014 |
| 6DVUB | 0.4528 | 0.4500 | 0.4481 | 0.4528 | 0.4434 | 0.4245 | 0.4528 | 0.4858 | 0.4764 | 0.4953 | 0.4556 | 0.4528 | 0.4623 |
| 6EMKB | 0.5317 | 0.5290 | 0.5267 | 0.5317 | 0.5317 | 0.5300 | 0.5333 | 0.5232 | 0.5175 | 0.5283 | 0.5238 | 0.5167 | 0.5275 |
| 6EWYA | 0.8246 | 0.8149 | 0.8047 | 0.8234 | 0.8244 | 0.8221 | 0.8259 | 0.7092 | 0.6940 | 0.7351 | 0.8075 | 0.7886 | 0.8159 |
| 6FDEA | 0.4412 | 0.4427 | 0.4412 | 0.4449 | 0.4456 | 0.4449 | 0.4485 | 0.4610 | 0.4559 | 0.4669 | 0.4442 | 0.4412 | 0.4449 |
| 6FPKA | 0.3585 | 0.3519 | 0.3491 | 0.3538 | 0.3538 | 0.3538 | 0.3538 | 0.3576 | 0.3538 | 0.3585 | 0.3585 | 0.3585 | 0.3585 |
| 6FTOA | 0.9731 | 0.9700 | 0.9692 | 0.9731 | 0.9669 | 0.9577 | 0.9769 | 0.9192 | 0.9077 | 0.9308 | 0.9723 | 0.9692 | 0.9769 |
| 6GWJB | 0.8304 | 0.8321 | 0.8304 | 0.8333 | 0.8256 | 0.8155 | 0.8304 | 0.8375 | 0.8125 | 0.8542 | 0.8446 | 0.8393 | 0.8482 |
| 6H2VA | 0.8173 | 0.8144 | 0.8113 | 0.8161 | 0.8161 | 0.8149 | 0.8173 | 0.7805 | 0.7656 | 0.8053 | 0.7909 | 0.7849 | 0.7981 |
| 6I9SA | 0.7286 | 0.7249 | 0.7219 | 0.7273 | 0.7273 | 0.7246 | 0.7286 | 0.6807 | 0.6564 | 0.7072 | 0.7257 | 0.7193 | 0.7313 |
| 6M3CB | 0.8491 | 0.8359 | 0.8208 | 0.8491 | 0.8443 | 0.8302 | 0.8538 | 0.7830 | 0.7547 | 0.8208 | 0.8434 | 0.8396 | 0.8443 |
| 6PCEB | 0.8750 | 0.8700 | 0.8679 | 0.8714 | 0.8493 | 0.8143 | 0.8679 | 0.8636 | 0.8464 | 0.8786 | 0.8764 | 0.8714 | 0.8786 |
| 6RBES | 0.6759 | 0.6762 | 0.6759 | 0.6776 | 0.6707 | 0.6586 | 0.6776 | 0.5928 | 0.5759 | 0.6034 | 0.6741 | 0.6707 | 0.6793 |
| 6RHVC | 0.8162 | 0.8035 | 0.8015 | 0.8074 | 0.8097 | 0.8074 | 0.8118 | 0.8321 | 0.8206 | 0.8500 | 0.8223 | 0.8147 | 0.8279 |
| 6RW4C | 0.9186 | 0.9137 | 0.9110 | 0.9167 | 0.8981 | 0.8920 | 0.9015 | 0.7742 | 0.7140 | 0.8352 | 0.9049 | 0.8977 | 0.9129 |
| 6T15h | 0.8971 | 0.8951 | 0.8922 | 0.8971 | 0.8706 | 0.8480 | 0.8873 | 0.5833 | 0.5539 | 0.6471 | 0.8922 | 0.8775 | 0.9020 |
| 6V92J | 0.4391 | 0.4404 | 0.4391 | 0.4435 | 0.4404 | 0.4391 | 0.4435 | 0.4013 | 0.3848 | 0.4196 | 0.4339 | 0.4261 | 0.4413 |
| 6VGJE | 0.6955 | 0.6981 | 0.6891 | 0.7019 | 0.6929 | 0.6923 | 0.6955 | 0.7289 | 0.7147 | 0.7372 | 0.6974 | 0.6891 | 0.7019 |
| 6VLZW | 0.5771 | 0.5825 | 0.5792 | 0.5854 | 0.5817 | 0.5729 | 0.5875 | 0.4383 | 0.4271 | 0.4563 | 0.5733 | 0.5625 | 0.5833 |
| 6WTID | 0.5278 | 0.5268 | 0.5227 | 0.5303 | 0.5288 | 0.5253 | 0.5303 | 0.4677 | 0.4621 | 0.4848 | 0.5253 | 0.5177 | 0.5303 |
| 6XAUA | 0.6282 | 0.6261 | 0.6218 | 0.6282 | 0.6256 | 0.6218 | 0.6303 | 0.6088 | 0.6029 | 0.6155 | 0.6252 | 0.6218 | 0.6282 |
| 6XYNA | 0.7737 | 0.7734 | 0.7722 | 0.7753 | 0.7747 | 0.7722 | 0.7769 | 0.7085 | 0.6535 | 0.7516 | 0.7712 | 0.7579 | 0.7769 |
| 6XYWB | 0.7468 | 0.7503 | 0.7484 | 0.7532 | 0.7484 | 0.7419 | 0.7565 | 0.5100 | 0.4855 | 0.5177 | 0.7423 | 0.7355 | 0.7468 |
| 6XYWR | 0.6077 | 0.6062 | 0.6022 | 0.6077 | 0.6080 | 0.6022 | 0.6168 | 0.5394 | 0.5292 | 0.5493 | 0.5934 | 0.5858 | 0.6022 |
| 6Y74A | 0.9535 | 0.9527 | 0.9506 | 0.9554 | 0.9535 | 0.9535 | 0.9535 | 0.9180 | 0.9021 | 0.9380 | 0.9387 | 0.9331 | 0.9428 |
| 6YBTu | 0.1903 | 0.1940 | 0.1884 | 0.1959 | 0.1922 | 0.1884 | 0.1940 | 0.3205 | 0.3153 | 0.3302 | 0.2112 | 0.2052 | 0.2201 |
| 6YI2A | 0.5559 | 0.5526 | 0.5493 | 0.5592 | 0.5579 | 0.5526 | 0.5625 | 0.5513 | 0.5395 | 0.5625 | 0.5579 | 0.5526 | 0.5658 |
| 7A1Gy | 0.7247 | 0.7165 | 0.7120 | 0.7215 | 0.7196 | 0.7120 | 0.7278 | 0.4462 | 0.4146 | 0.4873 | 0.7228 | 0.7215 | 0.7278 |
| 7AAZA | 0.7078 | 0.7083 | 0.7069 | 0.7095 | 0.7109 | 0.7095 | 0.7121 | 0.6776 | 0.6647 | 0.6853 | 0.6360 | 0.6164 | 0.6474 |
| 7AFRX | 0.3200 | 0.3240 | 0.3233 | 0.3250 | 0.3227 | 0.3217 | 0.3250 | 0.3213 | 0.3167 | 0.3233 | 0.3187 | 0.3133 | 0.3267 |
| 7AOEI | 0.6009 | 0.6096 | 0.6053 | 0.6140 | 0.5842 | 0.5482 | 0.6272 | 0.4561 | 0.4386 | 0.4737 | 0.6079 | 0.6009 | 0.6140 |
| 7B3JA | 0.4455 | 0.4482 | 0.4455 | 0.4545 | 0.4091 | 0.3864 | 0.4364 | 0.4500 | 0.4455 | 0.4591 | 0.4509 | 0.4409 | 0.4591 |
| 7DAWA | 0.7556 | 0.7517 | 0.7463 | 0.7556 | 0.7498 | 0.7434 | 0.7566 | 0.7131 | 0.6966 | 0.7313 | 0.7440 | 0.7397 | 0.7491 |
| 7EA8L | 0.5398 | 0.5493 | 0.5480 | 0.5500 | 0.5439 | 0.5418 | 0.5469 | 0.5272 | 0.5245 | 0.5306 | 0.5216 | 0.5153 | 0.5286 |
| 7EB9C | 0.8083 | 0.8075 | 0.8042 | 0.8125 | 0.7808 | 0.7042 | 0.8042 | 0.8025 | 0.7833 | 0.8292 | 0.8050 | 0.8000 | 0.8083 |
| 7EEBA | 0.3440 | 0.3444 | 0.3430 | 0.3459 | 0.3434 | 0.3421 | 0.3440 | 0.2963 | 0.2946 | 0.3033 | 0.3316 | 0.3275 | 0.3372 |
| 7EEBI | 0.4626 | 0.4641 | 0.4598 | 0.4655 | 0.4620 | 0.4612 | 0.4626 | 0.4667 | 0.4626 | 0.4698 | 0.4701 | 0.4655 | 0.4756 |
| 7EGAc | 0.6496 | 0.6520 | 0.6496 | 0.6555 | 0.6421 | 0.6299 | 0.6516 | 0.5114 | 0.5039 | 0.5197 | 0.6496 | 0.6476 | 0.6516 |
| 7EU4C | 0.4734 | 0.4724 | 0.4681 | 0.4761 | 0.4702 | 0.4654 | 0.4734 | 0.4372 | 0.4202 | 0.4521 | 0.4623 | 0.4601 | 0.4628 |
| 7EV1A | 0.6824 | 0.6783 | 0.6739 | 0.6824 | 0.6778 | 0.6751 | 0.6812 | 0.6442 | 0.6208 | 0.6606 | 0.6775 | 0.6739 | 0.6860 |
| 7JY6I | 0.8267 | 0.8298 | 0.8292 | 0.8308 | 0.8168 | 0.8100 | 0.8217 | 0.7763 | 0.7575 | 0.8025 | 0.7702 | 0.7617 | 0.7817 |
| AVERAGE | 0.6984 | 0.6970 | 0.6936 | 0.7004 | 0.6950 | 0.6876 | 0.7007 | 0.6538 | 0.6347 | 0.6716 | 0.6912 | 0.6847 | 0.6972 |

**Table S3.** The target-by-target performance of ATOMRefine, GNNRefine, and ModRefiner on 69 CASP14 regular targets evaluated by GDT-HA.

| Target ID | initial model | ATOMRefine | | | ATOMRefine_backbone | | | GNNRefine | | | ModRefiner | | |
| --- | --- | --- | --- | --- | --- | --- | --- | --- | --- | --- | --- | --- | --- |
|  |  | Mean | Minimum | Maximum | Mean | Minimum | Maximum | Mean | Minimum | Maximum | Mean | Minimum | Maximum |
| T1024 | 0.4687 | 0.4684 | 0.4661 | 0.4699 | 0.4684 | 0.4661 | 0.4719 | 0.4408 | 0.4284 | 0.4488 | 0.4400 | 0.4335 | 0.4457 |
| T1025 | 0.7558 | 0.7512 | 0.7490 | 0.7529 | 0.7558 | 0.7549 | 0.7568 | 0.7553 | 0.7500 | 0.7597 | 0.7545 | 0.7490 | 0.7588 |
| T1026 | 0.7483 | 0.7370 | 0.7295 | 0.7432 | 0.7514 | 0.7483 | 0.7551 | 0.7147 | 0.6952 | 0.7312 | 0.7620 | 0.7568 | 0.7671 |
| T1027 | 0.2470 | 0.2455 | 0.2440 | 0.2485 | 0.2411 | 0.2321 | 0.2470 | 0.2396 | 0.2366 | 0.2440 | 0.2443 | 0.2426 | 0.2470 |
| T1028 | 0.8570 | 0.8543 | 0.8510 | 0.8562 | 0.8563 | 0.8536 | 0.8579 | 0.8216 | 0.8082 | 0.8382 | 0.8373 | 0.8313 | 0.8450 |
| T1029 | 0.2540 | 0.2544 | 0.2540 | 0.2560 | 0.2556 | 0.2540 | 0.2580 | 0.2516 | 0.2480 | 0.2560 | 0.2540 | 0.2520 | 0.2560 |
| T1030 | 0.4212 | 0.4187 | 0.4167 | 0.4203 | 0.4222 | 0.4212 | 0.4231 | 0.4520 | 0.4377 | 0.4679 | 0.4161 | 0.4139 | 0.4185 |
| T1031 | 0.6553 | 0.6605 | 0.6579 | 0.6684 | 0.6584 | 0.6553 | 0.6605 | 0.6468 | 0.6368 | 0.6526 | 0.6569 | 0.6526 | 0.6632 |
| T1032 | 0.5603 | 0.5577 | 0.5544 | 0.5618 | 0.5612 | 0.5603 | 0.5632 | 0.5503 | 0.5456 | 0.5588 | 0.5520 | 0.5485 | 0.5574 |
| T1033 | 0.3425 | 0.3530 | 0.3500 | 0.3550 | 0.3485 | 0.3425 | 0.3575 | 0.3290 | 0.3250 | 0.3325 | 0.3470 | 0.3425 | 0.3525 |
| T1034 | 0.8526 | 0.8516 | 0.8478 | 0.8558 | 0.8526 | 0.8510 | 0.8542 | 0.8314 | 0.8221 | 0.8397 | 0.8375 | 0.8317 | 0.8413 |
| T1035 | 0.7598 | 0.7623 | 0.7574 | 0.7672 | 0.7642 | 0.7549 | 0.7696 | 0.7010 | 0.6814 | 0.7206 | 0.7711 | 0.7696 | 0.7770 |
| T1036s1 | 0.6900 | 0.7058 | 0.7033 | 0.7084 | 0.7033 | 0.6900 | 0.7088 | 0.5038 | 0.4438 | 0.5991 | 0.6654 | 0.6582 | 0.6690 |
| T1037 | 0.6479 | 0.6541 | 0.6522 | 0.6559 | 0.6512 | 0.6479 | 0.6541 | 0.6436 | 0.6318 | 0.6671 | 0.6467 | 0.6423 | 0.6498 |
| T1038 | 0.6921 | 0.6874 | 0.6842 | 0.6895 | 0.6918 | 0.6908 | 0.6934 | 0.6961 | 0.6829 | 0.7158 | 0.6724 | 0.6671 | 0.6816 |
| T1039 | 0.6351 | 0.6307 | 0.6273 | 0.6335 | 0.6363 | 0.6351 | 0.6366 | 0.6093 | 0.6025 | 0.6149 | 0.6379 | 0.6351 | 0.6413 |
| T1040 | 0.4288 | 0.4304 | 0.4269 | 0.4327 | 0.4292 | 0.4288 | 0.4308 | 0.4158 | 0.4115 | 0.4192 | 0.4235 | 0.4192 | 0.4288 |
| T1041 | 0.6932 | 0.6996 | 0.6973 | 0.7014 | 0.6957 | 0.6932 | 0.6973 | 0.6808 | 0.6736 | 0.6911 | 0.6886 | 0.6829 | 0.6911 |
| T1042 | 0.4819 | 0.4803 | 0.4792 | 0.4810 | 0.4806 | 0.4774 | 0.4828 | 0.4797 | 0.4683 | 0.4837 | 0.4770 | 0.4692 | 0.4810 |
| T1043 | 0.1081 | 0.1284 | 0.1284 | 0.1284 | 0.1247 | 0.1081 | 0.1301 | 0.1233 | 0.1233 | 0.1233 | 0.1287 | 0.1284 | 0.1301 |
| T1044 | 0.4755 | 0.4740 | 0.4726 | 0.4755 | 0.4733 | 0.4710 | 0.4753 | 0.4616 | 0.4505 | 0.4714 | 0.4893 | 0.4850 | 0.4939 |
| T1045s1 | 0.8360 | 0.8308 | 0.8263 | 0.8360 | 0.8354 | 0.8328 | 0.8377 | 0.8156 | 0.7955 | 0.8263 | 0.8192 | 0.8052 | 0.8295 |
| T1045s2 | 0.7877 | 0.7855 | 0.7816 | 0.7877 | 0.7828 | 0.7801 | 0.7877 | 0.7765 | 0.7349 | 0.7967 | 0.7883 | 0.7831 | 0.7937 |
| T1046s1 | 0.9271 | 0.9229 | 0.9167 | 0.9271 | 0.9299 | 0.9271 | 0.9340 | 0.8653 | 0.8576 | 0.8785 | 0.9257 | 0.9201 | 0.9306 |
| T1046s2 | 0.9078 | 0.9075 | 0.9043 | 0.9096 | 0.9078 | 0.9060 | 0.9113 | 0.9149 | 0.9113 | 0.9184 | 0.9025 | 0.8972 | 0.9096 |
| T1047s1 | 0.4100 | 0.4118 | 0.4088 | 0.4135 | 0.4102 | 0.4088 | 0.4123 | 0.3630 | 0.3412 | 0.3827 | 0.4073 | 0.4040 | 0.4111 |
| T1047s2 | 0.5025 | 0.5067 | 0.5049 | 0.5082 | 0.5043 | 0.5025 | 0.5066 | 0.4969 | 0.4515 | 0.5485 | 0.5043 | 0.5025 | 0.5074 |
| T1048 | 0.6594 | 0.6630 | 0.6594 | 0.6667 | 0.6290 | 0.5652 | 0.6594 | 0.6399 | 0.6196 | 0.6630 | 0.6609 | 0.6558 | 0.6667 |
| T1049 | 0.8601 | 0.8601 | 0.8563 | 0.8638 | 0.8604 | 0.8563 | 0.8638 | 0.8254 | 0.7985 | 0.8489 | 0.8489 | 0.8433 | 0.8563 |
| T1050 | 0.6261 | 0.6287 | 0.6268 | 0.6304 | 0.6267 | 0.6248 | 0.6284 | 0.6263 | 0.6137 | 0.6467 | 0.5968 | 0.5889 | 0.6065 |
| T1052 | 0.5018 | 0.5021 | 0.5000 | 0.5036 | 0.5020 | 0.5000 | 0.5036 | 0.4599 | 0.4366 | 0.4871 | 0.4715 | 0.4600 | 0.4781 |
| T1053 | 0.7697 | 0.7689 | 0.7663 | 0.7716 | 0.7717 | 0.7697 | 0.7731 | 0.7770 | 0.7678 | 0.7851 | 0.7492 | 0.7409 | 0.7553 |
| T1054 | 0.8392 | 0.8423 | 0.8409 | 0.8427 | 0.8357 | 0.8112 | 0.8479 | 0.8063 | 0.7605 | 0.8357 | 0.8381 | 0.8339 | 0.8409 |
| T1055 | 0.6803 | 0.6738 | 0.6619 | 0.6844 | 0.6783 | 0.6660 | 0.6844 | 0.7144 | 0.7049 | 0.7234 | 0.6939 | 0.6865 | 0.7029 |
| T1056 | 0.8402 | 0.8459 | 0.8447 | 0.8462 | 0.8388 | 0.8314 | 0.8462 | 0.8213 | 0.7988 | 0.8417 | 0.8364 | 0.8121 | 0.8506 |
| T1057 | 0.8303 | 0.8270 | 0.8242 | 0.8303 | 0.8285 | 0.8262 | 0.8303 | 0.8394 | 0.8343 | 0.8445 | 0.8148 | 0.7967 | 0.8232 |
| T1058 | 0.6479 | 0.6479 | 0.6452 | 0.6500 | 0.6487 | 0.6479 | 0.6493 | 0.6390 | 0.6164 | 0.6555 | 0.6434 | 0.6390 | 0.6493 |
| T1060s2 | 0.6288 | 0.6293 | 0.6246 | 0.6330 | 0.6271 | 0.6254 | 0.6288 | 0.5525 | 0.5227 | 0.5842 | 0.6151 | 0.6120 | 0.6178 |
| T1060s3 | 0.7947 | 0.8024 | 0.8008 | 0.8049 | 0.7963 | 0.7947 | 0.8008 | 0.7667 | 0.7520 | 0.7825 | 0.7716 | 0.7683 | 0.7744 |
| T1061 | 0.4745 | 0.4757 | 0.4739 | 0.4770 | 0.4750 | 0.4728 | 0.4770 | 0.4731 | 0.4679 | 0.4756 | 0.4683 | 0.4668 | 0.4705 |
| T1062 | 0.6143 | 0.6286 | 0.6214 | 0.6357 | 0.6229 | 0.6143 | 0.6357 | 0.6286 | 0.6214 | 0.6357 | 0.6386 | 0.6357 | 0.6429 |
| T1064 | 0.1348 | 0.1329 | 0.1324 | 0.1348 | 0.1353 | 0.1348 | 0.1373 | 0.1343 | 0.1299 | 0.1397 | 0.1333 | 0.1275 | 0.1348 |
| T1065s1 | 0.8298 | 0.8306 | 0.8277 | 0.8319 | 0.8298 | 0.8277 | 0.8319 | 0.8365 | 0.8298 | 0.8403 | 0.8235 | 0.8172 | 0.8277 |
| T1065s2 | 0.9209 | 0.9214 | 0.9158 | 0.9235 | 0.9214 | 0.9184 | 0.9235 | 0.8944 | 0.8724 | 0.9082 | 0.9163 | 0.9056 | 0.9260 |
| T1067 | 0.7998 | 0.8025 | 0.8020 | 0.8032 | 0.8004 | 0.7964 | 0.8054 | 0.7891 | 0.7805 | 0.8009 | 0.7914 | 0.7873 | 0.7975 |
| T1068 | 0.8827 | 0.8793 | 0.8743 | 0.8827 | 0.8861 | 0.8771 | 0.8911 | 0.8662 | 0.8464 | 0.8827 | 0.8869 | 0.8799 | 0.8897 |
| T1070 | 0.3341 | 0.3446 | 0.3434 | 0.3465 | 0.3361 | 0.3302 | 0.3449 | 0.3145 | 0.3117 | 0.3171 | 0.3323 | 0.3225 | 0.3434 |
| T1073 | 0.7288 | 0.7254 | 0.7246 | 0.7288 | 0.7297 | 0.7288 | 0.7331 | 0.7331 | 0.7246 | 0.7373 | 0.7152 | 0.7076 | 0.7288 |
| T1074 | 0.7519 | 0.7546 | 0.7519 | 0.7576 | 0.7459 | 0.7273 | 0.7614 | 0.7216 | 0.7102 | 0.7386 | 0.7561 | 0.7500 | 0.7633 |
| T1076 | 0.8949 | 0.8884 | 0.8866 | 0.8903 | 0.8925 | 0.8884 | 0.8949 | 0.8575 | 0.8259 | 0.8833 | 0.8779 | 0.8708 | 0.8833 |
| T1078 | 0.8992 | 0.9186 | 0.9167 | 0.9205 | 0.8950 | 0.8876 | 0.8992 | 0.8942 | 0.8798 | 0.9167 | 0.8985 | 0.8837 | 0.9109 |
| T1079 | 0.7469 | 0.7486 | 0.7443 | 0.7505 | 0.7476 | 0.7464 | 0.7490 | 0.7439 | 0.7360 | 0.7474 | 0.7426 | 0.7370 | 0.7495 |
| T1080 | 0.6523 | 0.6659 | 0.6635 | 0.6673 | 0.6635 | 0.6523 | 0.6729 | 0.4932 | 0.4680 | 0.5244 | 0.6274 | 0.5733 | 0.6748 |
| T1082 | 0.8000 | 0.8317 | 0.8267 | 0.8333 | 0.8000 | 0.7767 | 0.8267 | 0.8267 | 0.8167 | 0.8367 | 0.8313 | 0.8267 | 0.8367 |
| T1083 | 0.7391 | 0.7397 | 0.7337 | 0.7446 | 0.7331 | 0.7228 | 0.7391 | 0.7679 | 0.7500 | 0.7880 | 0.7386 | 0.7310 | 0.7473 |
| T1084 | 0.8803 | 0.8838 | 0.8732 | 0.8908 | 0.8810 | 0.8768 | 0.8873 | 0.8289 | 0.8134 | 0.8592 | 0.8768 | 0.8732 | 0.8803 |
| T1087 | 0.9167 | 0.9188 | 0.9140 | 0.9220 | 0.9140 | 0.9032 | 0.9194 | 0.8532 | 0.8280 | 0.8844 | 0.9167 | 0.9113 | 0.9247 |
| T1088 | 0.5030 | 0.5036 | 0.5030 | 0.5045 | 0.5048 | 0.5030 | 0.5075 | 0.5054 | 0.4970 | 0.5166 | 0.4967 | 0.4940 | 0.5000 |
| T1089 | 0.9244 | 0.9244 | 0.9231 | 0.9257 | 0.9224 | 0.9171 | 0.9251 | 0.8936 | 0.8813 | 0.9072 | 0.9113 | 0.9005 | 0.9145 |
| T1091 | 0.4747 | 0.4778 | 0.4768 | 0.4790 | 0.4761 | 0.4747 | 0.4774 | 0.4860 | 0.4709 | 0.4946 | 0.4534 | 0.4423 | 0.4688 |
| T1092 | 0.5041 | 0.5058 | 0.5047 | 0.5065 | 0.5029 | 0.5023 | 0.5041 | 0.3369 | 0.3081 | 0.4085 | 0.4800 | 0.4707 | 0.4853 |
| T1093 | 0.4642 | 0.4714 | 0.4686 | 0.4726 | 0.4663 | 0.4642 | 0.4674 | 0.4372 | 0.4209 | 0.4475 | 0.4262 | 0.4189 | 0.4364 |
| T1094 | 0.4840 | 0.4941 | 0.4923 | 0.4974 | 0.4858 | 0.4830 | 0.4897 | 0.4893 | 0.4845 | 0.4990 | 0.4736 | 0.4680 | 0.4788 |
| T1095 | 0.4730 | 0.4797 | 0.4780 | 0.4811 | 0.4755 | 0.4730 | 0.4776 | 0.4700 | 0.4626 | 0.4784 | 0.4625 | 0.4541 | 0.4688 |
| T1096 | 0.3410 | 0.3438 | 0.3426 | 0.3448 | 0.3429 | 0.3410 | 0.3459 | 0.3436 | 0.3377 | 0.3568 | 0.3171 | 0.3126 | 0.3203 |
| T1098 | 0.2340 | 0.2323 | 0.2317 | 0.2328 | 0.2345 | 0.2340 | 0.2351 | 0.2348 | 0.2334 | 0.2368 | 0.2330 | 0.2305 | 0.2374 |
| T1099 | 0.5691 | 0.5737 | 0.5705 | 0.5824 | 0.5721 | 0.5691 | 0.5771 | 0.5532 | 0.5465 | 0.5598 | 0.5585 | 0.5426 | 0.5652 |
| T1100 | 0.5744 | 0.5787 | 0.5767 | 0.5813 | 0.5787 | 0.5744 | 0.5805 | 0.6209 | 0.5966 | 0.6480 | 0.5841 | 0.5744 | 0.5982 |
| T1101 | 0.7264 | 0.7259 | 0.7231 | 0.7281 | 0.7279 | 0.7264 | 0.7315 | 0.7207 | 0.7104 | 0.7281 | 0.7289 | 0.7239 | 0.7332 |
| AVERAGE | 0.6391 | 0.6415 | 0.6386 | 0.6442 | 0.6396 | 0.6342 | 0.6440 | 0.6202 | 0.6056 | 0.6360 | 0.6331 | 0.6261 | 0.6397 |

**Table S4.** Performance of ATOMRefine, GNNRefine, and ModRefiner on seven CASP14 refinement targets with different starting models evaluated by GDT-HA.

| **Target**  **ID** | **Residues** | **Classification** | **Initial**  **model type** | **Start**  **model** | **ATOMRefine** | **GNNRefine** | **ModRefiner** |
| --- | --- | --- | --- | --- | --- | --- | --- |
| R1040v1 | 130 | FM | AF2 | **54.88** | 54.62 | 50.00 | 53.08 |
| R1040v2 |  |  | TS435_2 | 29.30 | 30.77 | **32.31** | 30.96 |
| R1041v1 | 242 | FM | AF2 | 70.33 | 70.33 | 70.33 | **71.00** |
| R1041v2 |  |  | TS031_1 | 40.67 | **40.89** | 40.44 | 40.22 |
| R1042v1 | 276 | FM | TS403_1 | 34.68 | **34.88** | 34.78 | 34.48 |
| R1042v2 |  |  | AF2 | 62.70 | **63.00** | 62.00 | 62.20 |
| R1043v1 | 148 | FM | TS403_1 | **44.09** | 43.92 | 43.75 | 43.92 |
| R1043v2 |  |  | AF2 | 64.86 | **65.54** | 63.34 | 64.86 |
| R1053v1 | 171 | FM/TBM | TS042_5-D2 | 52.63 | 52.78 | **53.07** | 52.78 |
| R1053v2 |  |  | AF2 | 79.53 | **80.26** | 73.10 | 79.53 |
| R1067v1 | 221 | TBM-hard | TS473_3 | 45.48 | 45.14 | **45.48** | 45.14 |
| R1067v2 |  |  | AF2 | 78.85 | **79.19** | 78.51 | 78.86 |
| R1074v1 | 132 | FM | AF2 | 78.22 | **78.60** | 76.33 | 78.03 |
| R1074v2 |  |  | TS140_5 | **35.42** | **35.42** | **35.42** | 35.80 |
| Average | | | AF2 | 69.91 | **70.22** | 67.66 | 69.62 |
|  |  |  | CASP14 other groups | 40.32 | 40.54 | **40.75** | 40.47 |

| **a. AF2 start model** | | | | | | |
| --- | --- | --- | --- | --- | --- | --- |
| **Target ID** | **Residues in refinement target** | **Classification** | **Start model GDT-HA** | **ATOMRefine GDT-HA** | **GNNRefine GDT-HA** | **ModRefiner GDT-HA** |
| R1040v1 | 130 | FM | **54.88** | 54.62 | 50.00 | 53.08 |
| R1041v1 | 242 | FM | 70.33 | 70.33 | 70.33 | **71.00** |
| R1042v2 | 276 | FM | 62.70 | **63.00** | 62.00 | 62.20 |
| R1043v2 | 148 | FM | 64.86 | **65.54** | 63.34 | 64.86 |
| R1053v2 | 171 | FM/TBM | 79.53 | **80.26** | 73.10 | 79.53 |
| R1067v2 | 221 | TBM-hard | 78.85 | **79.19** | 78.51 | 78.62 |
| R1074v1 | 132 | FM | 78.22 | **78.6** | 76.33 | 78.03 |
| **b. Other CASP14 start model** | | | | | | |
| **Target ID** | **Residues in refinement target** | **Classification** | **Start model GDT-HA** | **ATOMRefine GDT-HA** | **GNNRefine GDT-HA** | **ModRefiner GDT-HA** |
| R1040v2 | 130 | FM | 29.3 | 30.77 | **32.31** | 30.96 |
| R1041v2 | 242 | FM | 40.67 | **40.89** | 40.44 | 40.22 |
| R1042v1 | 276 | FM | 34.68 | **34.88** | 34.78 | 34.48 |
| R1043v1 | 148 | FM | **44.09** | 43.92 | 43.75 | 43.92 |
| R1053v1 | 171 | FM/TBM | 52.63 | **52.78** | 53.07 | 52.78 |
| R1067v1 | 221 | TBM-hard | **45.48** | 45.14 | **45.48** | 45.14 |
| R1074v2 | 132 | FM | **35.42** | **35.42** | **35.42** | 35.80 |

**Table S5.** Performance of ATOMRefine, GNNRefine, and ModRefiner on seven CASP14 refinement targets with different starting models. **(a)** AF2 start model; **(b)** other non-AF2 CASP14 start model. The start and refined models are evaluated by GDT-HA.


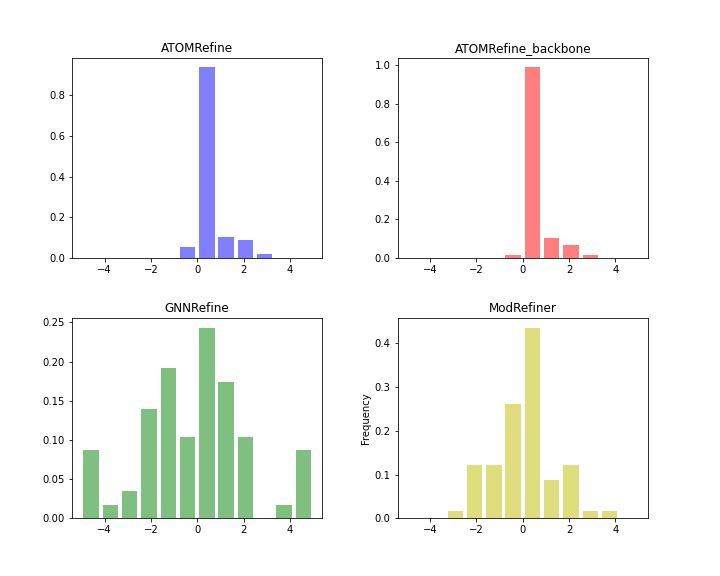


Density

Fig. S1. The distribution (histogram) of quality change (ΔGDT-HA score) of refined models of ATOMRefine, GNNRefine, ModRefiner, and ATOMRefine_backbone with respect to the initial models on the CASP14 test set.  The positive value means the model quality after refinement improves from the starting model and the negative value means the model quality decreases. To obtain a better visualization, ΔGDT-HA is clipped into the range [-5, 5].
